# Insights from Modulation-Excitation Spectroscopy into the Role of Pt Geometrical Sites in the WGS Reaction

**DOI:** 10.1021/acsami.4c21397

**Published:** 2025-02-22

**Authors:** Tathiana M. Kokumai, Larissa E. R. Ferreira, Guilherme B. Strapasson, Lea Pasquale, Liberato Manna, Massimo Colombo, Daniela Zanchet

**Affiliations:** † Institute of Chemistry, University of Campinas, Campinas, SP 13083-970, Brazil; ‡ Brazilian Synchrotron Light Laboratory, CNPEM, Campinas, SP 13083-100, Brazil; § Nanochemistry Department, Italian Institute of Technology, Genoa, GE 16163, Italy

**Keywords:** modulation-excitation spectroscopy, Pt catalysts, water−gas shift reaction, infrared Fourier transform spectroscopy

## Abstract

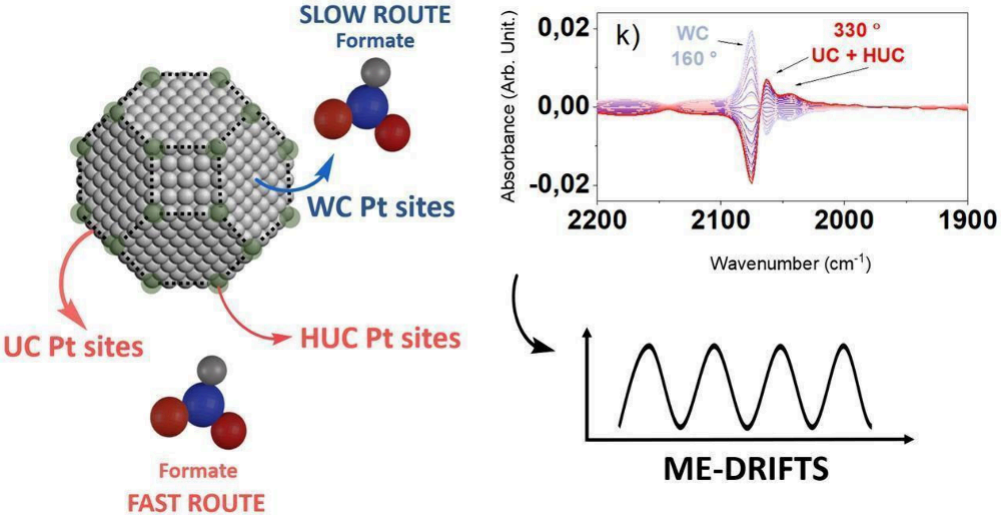

Modulation-excitation spectroscopy coupled to diffuse reflectance infrared Fourier transform spectroscopy (ME-DRIFTS) was explored in this work to obtain valuable insights into the structure–reactivity relations in nanostructured Pt catalysts for the water–gas shift (WGS) reaction. By using model Pt catalytic systems composed of colloidal Pt nanoparticles (NPs) deposited on CeO_2_ (i.e., reducible) and SiO_2_ (i.e., nonreducible) supports, it was possible to probe distinct Pt active sites and correlate them to the reaction intermediates and pathways. The analysis revealed that PtNPs/SiO_2_ favored the participation of well-coordinated (WC) and under-coordinated (UC) Pt sites in the reaction mechanism. In contrast, on PtNPs/CeO_2_/SiO_2_, the additional involvement of highly under-coordinated (HUC) Pt sites was also observed. Additionally, both fast and slow formate species were identified as active intermediates on the surface of the PtNPs/CeO_2_/SiO_2_ catalyst by ME-DRIFTS. More importantly, the faster reaction pathway was correlated to HUC and UC Pt sites, while the slower route was associated with WC Pt sites. Carbonates, on the other hand, were spectators. ME-DRIFTS experimentally demonstrate differences in the participation of Pt active sites according to the support, the involvement of interfacial sites, and the correlation of Pt local coordination to the surface intermediates in the WGS reaction.

## 1 Introduction

It is challenging and crucial to develop accessible methodologies to probe heterogeneous catalysts under reaction conditions with surface sensitivity and time resolution to detect and distinguish short-lived intermediates and active sites. A wide-use and valuable tool that contributes to the comprehension of the catalytic pathways and catalyst surface properties is *in situ* diffuse reflectance infrared Fourier transform spectroscopy (DRIFTS). In a typical *in situ* DRIFTS experiment, the catalyst is usually pretreated (ex., reduced under H_2_) and exposed to reactants, temperature, and pressure, and the species present on the surface are characterized by their vibrational modes. One of the main drawbacks of conventional *in situ* DRIFTS experiments is the inherent difficulty distinguishing active surface reaction intermediates, i.e., species that participate in the reaction mechanism, from spectator species. More elaborate combinations, such as using isotopic label experiments, have provided valuable insights into the reaction mechanism of several reactions. An accessible alternative has been coupling DRIFTS with modulation-excitation spectroscopy (ME-DRIFTS) and phase sensitive detection (PSD), which has the power to distinguish active surface reaction intermediates from spectator species and to enhance the signal-to-noise ratio of the spectra significantly.^1−5^ Previous reports show that ME-DRIFTS has been applied with success in different reactions, such as CO oxidation,^6,7^ NH_3_ synthesis,^8^ toluene oxidation,^9^ and water gas shift (WGS) reaction,^4,10^ revealing various aspects of the reaction mechanisms and catalytic sites.

As a case study, the WGS reaction is an interesting example of the intense debate in the literature about identifying intermediaries and spectators and their dependence on the reaction conditions and catalyst nature based on theoretical and experimental results using different techniques. In the WGS reaction, CO reacts with H_2_O to produce CO_2_ and H_2_, eq 1.
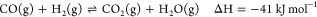
1

Low-temperature WGS catalysts usually comprise Pt-, Au-, Pd-, or Cu-supported systems,^9,11−15^ that may or may not present other promoters (e.g., alkali, oxides).^16,17^ There is clear evidence that some supports also have an active role in the WGS reaction mechanism, demonstrated mainly by the high activity of supported catalysts based on reducible metal oxides, e.g., TiO_2_ and CeO_2_.^11,18,19^ The enhanced catalytic performance can be attributed to the availability of bulk and surface oxygen vacancies and their effect on the oxygen storage capacity; metal–support interactions; promotion of spillover species (*CO and intermediaries), e.g., from interfacial to metallic sites; and thermal stability.^20,21^ Other properties, such as the loading, particle size, exposed crystal facets, and metal charge transfer, have been investigated.^20,22−31^

Several reaction mechanisms for the WGS reaction have been proposed, depending on the catalyst and the reaction conditions. The “redox” mechanism is generally accepted for metal-supported over-reducible metal oxides, according to the reaction temperature.^32,33^ This route involves the oxidation of adsorbed *CO by oxygen from the oxide lattice with the formation of an oxygen vacancy; this vacancy is regenerated in the presence of H_2_O without forming intermediaries.^34−38^ On the other hand, for nonreducible metal oxide supports, e.g., SiO_2_ or Al_2_O_3_, the WGS proceeds by the “associative” mechanism, where *H and *OH adsorbed species react with adsorbed *CO producing intermediates such as carboxylates (*COO^–^), formates (*HCOO^–^), and carbonates (*CO_3_^δ−^), which decompose into CO_2(g)_ and H_2(g)_.^32−34,39^ Additionally, the associative mechanism can also occur by a redox generation step (i.e., employing reducible metal oxide supports), in which the *OH groups consumed to form the intermediates would generate an oxygen vacancy to be restored by water dissociation.^35,40,41^

The nature of active sites can be correlated with the geometrical features, in which exposed atoms can present varied local coordination: highly under-coordinated sites (HUC, coordination lower than 6 atoms), under-coordinated sites (UC, coordination of 6–7), and well-coordinated sites (WC, coordination of 8–9 atoms). Considering a truncated cuboctahedron particle model, these sites can be associated with corners (HUC), edges (UC), and terrace sites (WC), and the number of low-coordination sites (i.e., HUC and UC) decreases with the increase in particle size.^42−44^ Moreover, in a metal-supported system, the metal atoms at the edges and corners are in close contact with the support (i.e., interfacial sites). An elegant example employing *in situ* transmission electron microscopy (TEM) of a Pt/CeO_2_ system demonstrated that Pt–Pt bonds are weakened under a CO exposure at 200 °C, resulting in dynamic Pt atoms. Under WGS reactional conditions, the Pt atoms became more stabilized/localized, except those at the metal–support interface.^12^ This study demonstrated that the Pt interfacial sites are dynamically mobile and correlated with highly active sites. Previous reports on Pt/CeO_2_ catalysts also demonstrated that the Pt^δ+^–O_v_–Ce^3+^ interface was responsible for the activation of CO and H_2_O and further dissociation of H_2_O into *OH and *H, along with localized distortions on the Pt particles that facilitate CO mobility.^12,18,35,40,45^ Yu et al.^45^ showed that the metal–support interaction also depends on the CeO_2_ exposed facet: Pt clusters were embedded within 3–4 atomic layers of the CeO_2_ lattice in the case of the (110) facet, promoting a stronger charge transfer and enhancing the formation of Pt^δ+^–O_v_–Ce^3+^; this phenomenon was absent in the (100) facet. Another study investigated a 0.5 wt % Pt/Ce_0.5_La_0.5_O_2−δ_ catalyst during the WGS reaction by isotopic transient DRIFTS and suggested that HUC and UC Pt sites were involved in the formation of active reaction intermediates, whereas WC Pt sites were not.^46^ Although several studies shared a consensus on the interfacial sites’ role in the WGS reaction, the reaction mechanism discrepancies between true intermediates and spectators depending on the catalyst nature and reaction conditions emphasize the complexity of the WGS reaction pathways.

In this work, we explored ME-DRIFTS to study the impact of the support (i.e., reducible or nonreducible) and the role of different Pt geometrical sites on the WGS reaction. Pt/SiO_2_ catalysts with and without CeO_2_ as a promoter were designed; colloidal Pt nanoparticles were prepared to avoid significant variations in the shape, size, and geometrical features of the metallic phase and used to probe the role of the CeO_2_ phase. The ME-DRIFTS spectra shed light on the differences in the active Pt sites of PtNPs/SiO_2_ and PtNPs/CeO_2_/SiO_2_, the participation of metal-oxide interfacial sites, and the correlation of Pt local coordination to the surface intermediates in the WGS reaction.

## 2 Experimental Section

### 2.1 Catalyst Preparation

Silica Aerosil 380 from Evonik, with a reported surface area of 350–410 m^2^ g^–1^, was used as support. All other materials were purchased from Sigma-Aldrich and employed without additional purification.

### 2.2 Synthesis of Colloidal Pt NPs

Colloidal Pt NPs were obtained according to the procedure described in the literature.^34^ The precursor Pt(acac)_2_ (0.2 mmol) was added under stirring to a round-bottomed flask containing trioctylamine (22.9 mmol), followed by oleylamine (2.0 mmol) and oleic acid (8.0 mmol) addition. The mixture was kept under vacuum at room temperature for 5 min. Trioctylphosphine (0.1 mmol) was injected, and the mixture was heated to 120 and 15 °C min^–1^ for 30 min. The atmosphere was changed to N_2_, and the flask was quickly heated to 250 °C (40 °C min^–1^) and held for 30 min. After being cooled to room temperature, the NPs were precipitated with a mixture of 15 mL of isopropanol and 20 mL of methanol and centrifuged at 6500 rpm for 3 min (this procedure was repeated twice). Finally, Pt NPs were collected and redispersed in hexane.

### 2.3 Synthesis of Colloidal CeO_2_ NPs (5 nm) and Preparation of the Supports

CeO_2_ NPs with a mean size of 5 nm were synthesized as described by Lee et al.^47^ The precursor Ce(NO_3_)_3_·6H_2_O (1 mmol) was added to a round-bottomed flask containing 5.0 mL of 1-octadecene under stirring. Oleylamine (3 mmol) was added, and the system was purged with vacuum and N_2_ 3 times and left under stirring in a N_2_ atmosphere. The mixture was heated following the protocol: 80 °C, 10 °C min^–1^, soak time of 30 min; 260 °C, 10 °C min^–1^, soak time of 2 h. The mixture was quickly cooled (with a stream of compressed air outside the flask) to 60 °C, precipitated with a 1:1 (v/v) acetone:methanol (25 mL) solution, and centrifuged at 4500 rpm for 30 min to remove the excess of ligands and 1-octadecene. The precipitation/washing was repeated 5 times. Finally, the CeO_2_ NPs were redispersed in hexane.

A CeO_2_/SiO_2_ support was prepared by depositing the colloidal CeO_2_ NPs on silica using a wet impregnation method. CeO_2_ NPs were deposited over the silica support with a nominal loading of 12% wt. of CeO_2_. A 0.5 g portion of silica was suspended in 30 mL of toluene under stirring. The corresponding volume of the initial dispersion of CeO_2_ NPs was diluted in 20 mL of toluene and added to the silica suspension. The mixture was kept under stirring for ∼19 h and centrifuged at 4500 rpm for 10 min, and the solvent was removed in a rotating evaporator. The solid was dried overnight in an oven at 70 °C, calcined at 450 °C, 5 °C min^–1^, for 1 h under synthetic air flow (80 mL min^–1^) to remove the organic capping ligands. The support was labeled CeO_2_/SiO_2_ and compared to bare SiO_2_.

### 2.4 Preparation of Catalysts by Pt NPs Deposition

The catalysts were prepared by the deposition of the colloidal Pt NPs over the supports using a wet impregnation methodology, following the same procedure as that described for the deposition of the CeO_2_ NPs. The nominal Pt loading was 2% wt. Pt for both supports (SiO_2_ and CeO_2_/SiO_2_), followed by a calcination step at 450 °C, 5 °C min^–1^, for 1 h under synthetic air flow (80 mL min^–1^), to remove the residual organic ligands. The resulting samples were named PtNPs/SiO_2_ and PtNPs/CeO_2_/SiO_2_.

### 2.5 Characterization

The catalysts were characterized by X-ray diffraction (XRD) on a Shimadzu XRD7000 equipped with a Cu target (Kα = 1.5406 Å) and a crystal analyzer, operating at 40 kV and 30 mA. In the case of the colloidal Pt NPs, the dispersion on hexane was deposited on a Si substrate to reduce the background signal. Cerium loadings were obtained by X-ray fluorescence (XRF) in a Shimadzu XRF1800. Pt loadings were determined by inductively coupled plasma optical emission spectroscopy (ICP-OES) using an iCAP 6000 Thermo Scientific spectrometer at the Italian Institute of Technology (IIT). The powder catalysts were digested in HCl/HNO_3_ 3/1 (v/v) for 1 h at 250 °C, followed by dilution with deionized water (14 mS), and filtered using a PTFE filter (45 μm) before each measurement. For each sample, the procedure was repeated three times. The colloidal NPs were analyzed by transmission electron microscopy (TEM) on a TEM-MSC JEOL 2100 200 kV instrument at the Brazilian Nanotechnology National Laboratory at the Brazilian Center for Research in Energy and Materials (LNNano-CNPEM). The TEM images for powder samples were obtained by a JEOL JEM-1400 Plus 120 kV instrument (IIT).

DRIFTS spectra were acquired on a Vertex 70 infrared spectrometer (Bruker Optics) equipped with a DRIFT cell (Praying Mantis, Harrick) and a liquid-nitrogen-cooled MCT detector. The cell was composed of a dome with two ZnS_2_ windows, and an additional glass window for sample observation. The temperature was measured with a thermocouple installed at the center of the catalyst bed. Before the experiments, the catalyst was reduced *in situ* at 400 °C for 1 h under H_2_ flow (25 mL min^–1^) and cooled to the reaction temperature. For CO adsorption experiments, the catalyst was exposed for 10 min to a flow of 1%CO/He, 80 mL min^–1^, and the spectra were collected at room temperature every 10 s for the first 2 min (which already showed saturation of Pt surface), then every 30 s for the following 5 min. For CO desorption, the flow was switched to He (80 mL min-1) with a heating rate of 10 °C min^–1^ to 400 °C while the spectra were collected under the same conditions. After 1 h at 400 °C under He, when necessary, the flow was changed to H_2_, 25 mL min^–1^, and spectra were acquired to observe the desorption of CO from the Pt surface.

For ME-DRIFTS experiments, two gas flow mixtures could be alternatively allowed inside the DRIFT cell through a gas supply system equipped with mass flow controllers (Bronkhorst) and a two-position valve actuator (VICI-Valco). This valve allowed for a quick and periodic switch between the two gas mixtures with a desired frequency. Gas exiting the cell was analyzed online through a mass spectrometer (Omnistar, Pfeiffer). Spectra were reported in absorbance units. A background spectrum was collected in He at each temperature before the catalyst exposure to the analysis gas. After *in situ* reduction, at 250 °C, the catalyst was exposed alternately to CO+H_2_O/CO (1 mL min^–1^ CO and 3 mL min^–1^ of H_2_O, balance He and 1 mL·min^–1^ CO, balance He, with total flows of 103 mL min^–1^). Thus, both streams kept the same total flows and the same CO concentration. Figure S1 illustrates the experiment and shows that the periodic stimulation (in this case, the alternating gas flows) generates a periodic response of the probed species captured by spectra acquisition. During a modulation period comprising one cycle with the two alternating gas atmospheres, CO+H_2_O/CO (period T = 300 s, frequency, ω = 3.3 mHz), 60 consecutive spectra were collected at a resolution of 4 cm^–1^. Thus, two sets of 30 spectra each were acquired for each gas flow in one cycle. Each spectrum is a snapshot of the catalyst surface at a given time in one cycle (for example, at *tx* and *ty* seconds). To increase the signal-to-noise ratio, the full cycle was repeated 22 times; only the last 12 were averaged to take into account the time required for the system to reach a quasi-steady state condition (stabilization time). The resulting averaged spectra, each at a given time (*tx*, *ty*, in the time domain), were processed into phase-resolved spectra using phase-sensitive detection (PSD), as detailed in the Supporting Information. Other conditions (temperature and gas atmospheres) were initially tested and are presented in the Supporting Information.

### 2.6 Catalytic Tests

A WGS reaction was performed in a fixed bed quartz reactor (i.d. nine mm) operating at atmospheric pressure. Typically, 25 mg of catalyst was mixed with 75 mg of ground quartz as a diluent. Before the reaction, the samples were reduced under 35 mL·min^–1^ of H_2_ at 400 °C for 1 h. The catalyst was cooled to 250 °C, and the WGS reaction was performed with a 1:3 CO: H_2_O (v/v) feed ratio and total (wet) flow of 115 mL min^–1^, 4.3% v/v CO. Unreacted steam was condensed before the outlet stream reached the gas chromatograph system, which consisted of an Agilent CG 7890 instrument equipped with a TCD detector. The reaction was performed at temperatures of 250, 300, 350, and 400, with 5 measurements at each temperature. CO_2_ production rates (mol CO_2_·mol Pt sites^–1^·min^–1^) were obtained by the molar flow of CO_2_ produced divided by the amount of exposed Pt sites obtained by extended X-ray absorption fine structure (EXAFS) analysis of Pt NPs at Pt-L_3_ edge, see Supporting Information for details and Figure S2.

## 3 Results and Discussion

### 3.1 Catalysts’ Synthesis and Initial Characterization

**Figure 1:**
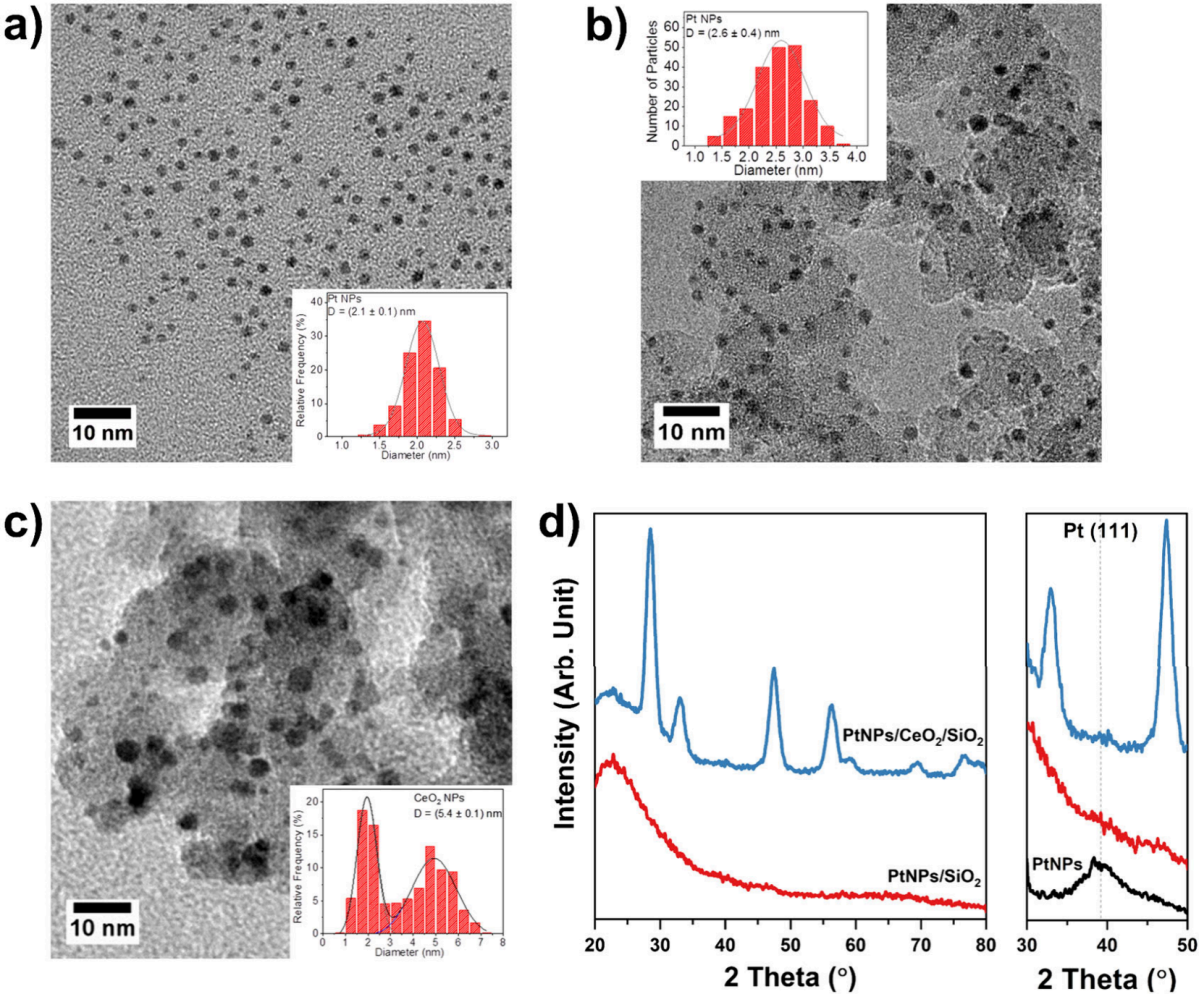
(a–c) TEM images and corresponding size distributions of (a) colloidal Pt NPs, (b) PtNPs/SiO_2_, and (c) PtNPs/CeO_2_/SiO_2_. (d) XRD profiles of colloidal Pt NPs (black) and the final catalysts PtNPs/SiO_2_ (red) and PtNPs/CeO_2_/SiO_2_ (blue); dashed line indicates the position of (111) reflection of Pt fcc structure.

ICP and XRF measurements of Pt and Ce, respectively, confirmed loadings close to the nominal amounts for PtNPs/SiO_2_ (2.0 wt % Pt) and PtNPs/CeO_2_/SiO_2_ (2.2 wt % Pt and 11.0 wt % CeO_2_). Figure 1a shows a representative TEM image of the as-synthesized colloidal PtNPs, composed of spherical particles with a mean size of (2.1 ± 0.1) nm and narrow size distribution. Pt NPs retained a similar mean size in PtNPs/SiO_2_ (2.6 ± 0.4) nm (Figure 1b), demonstrating that the calcination step did not significantly impact the metal dispersion. In the case of PtNPs/CeO_2_/SiO_2_, the distinction between CeO_2_ and Pt NPs in the TEM images is not straightforward due to the similarities in size and contrast (Figure 1c). Nevertheless, the histogram revealed two distinct populations of NPs, a smaller one with a mean particle size centered at (2.0 ± 0.1) nm and another one at (4.9 ± 0.1) nm, in agreement with the presence of colloidal Pt and CeO_2_ NPs, respectively. Hence, it can be assumed that Pt NPs are similar in both catalysts, assuring that size and shape are comparable. XRD patterns of PtNPs, PtNPs/SiO_2_, and PtNPs/CeO_2_/SiO_2_ (Figure 1d) corroborate the nanosized domains of PtNPs. PtNPs presented a broad (111) reflection with an estimated mean crystallite size of 1.9 nm, in agreement with the TEM results (Figure 1a). PtNPs/SiO_2_ presented a broad feature at 2θ = 30°, related to the amorphous silica, while PtNPs/CeO_2_/SiO_2_ presented reflections matching the CeO_2_ fluorite structure with a mean crystallite size of 5 nm, in accordance with the mean size of the CeO_2_ NPs obtained by XRD and TEM (Figures S3, S4). The absence of the Pt (111) reflection in both supported catalysts confirmed no sintering of the Pt NPs during the preparation of the catalysts, in agreement with the TEM images (Figure 1b,c).

**Figure 2:**
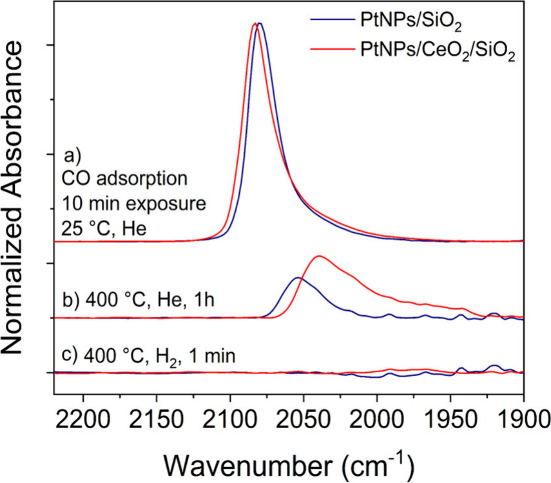
CO-DRIFTS spectra of the catalysts (a) after exposure to a CO flow (1% CO/He v/v) for 10 min, (b) after desorption under a He flow at 400 °C for 1 h, and (c) after 1 min under a H_2_ flow at 400 °C.

CO-DRIFTS spectra of prereduced PtNPs/SiO_2_ and PtNPs/CeO_2_/SiO_2_ during CO adsorption and desorption are presented in Figure 2. CO adsorption spectra at room temperature (Figure 2a) presented one main asymmetric band with similar width and maxima around 2080 cm^–1^ for both catalysts, associated with CO linearly bound to metallic WC Pt atoms.^48−50^ The slight shift of PtNPs/CeO_2_/SiO_2_ to higher wavenumbers suggests a stronger metal–support interaction. Contributions related to HUC and UC sites (i.e., 2040–2070 cm^–1^) were highlighted by the deconvolution of the spectra (Figure S5) with WC:UC:HUC area ratios of 56:26:18 and 51:26:23 for PtNPs/SiO_2_ and PtNPs/CeO_2_/SiO_2_, respectively. The similarity of the spectra within the resolution indicates that CO molecules were bound to similar Pt sites, and therefore, intrinsic PtNPs properties, such as size, distribution of WC, HUC, and UC Pt sites, and electronic properties, are comparable between the catalysts. The metallic nature of Pt was confirmed by XANES and EXAFS measurements of PtNPs/CeO_2_/SiO_2_ (Figure S2, Table S1).

Figure 2b shows that after CO desorption under a He flow at 400 °C for 1 h, both catalysts presented contributions with the maximum in the 2040–2053 cm^–1^ region, along with the intense variation in the signal background caused by the high temperature of the DRIFTS cell. These contributions at lower wavenumber are mainly attributed to CO linearly bound to HUC Pt sites.^48−51^ The fact that CO could not be completely desorbed under these conditions illustrates the stronger interaction with these sites; the contributions related to the WC sites vanished. The HUC Pt sites present a higher available electronic density, resulting in stronger CO-Pt bonds, weakening the C–O bond, and causing its vibration to shift to lower wavenumbers. The surface of PtNPs could only be cleaned from CO by flowing H_2_ at 400 °C, which quickly replaced CO molecules, as observed in Figure 2c.

### 3.2 WGS Reaction

**Figure 3:**
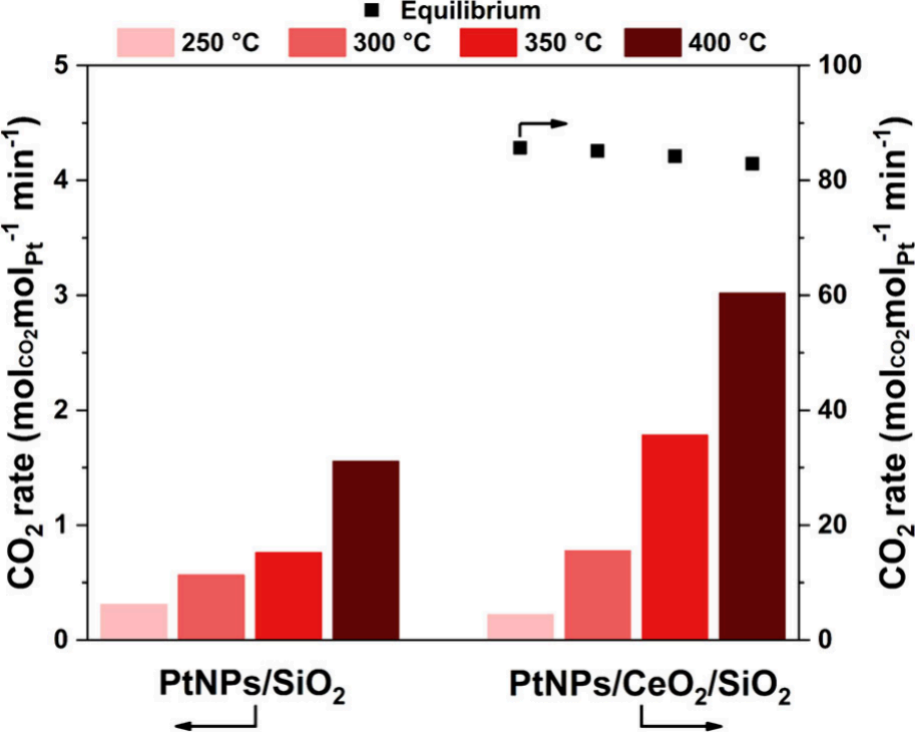
WGS activity as a function of temperature represented as the CO_2_ rate (mol of CO_2_ produced per mol of exposed Pt sites per minute). Square symbols represent the equilibrium of the CO_2_ rate. Conditions: CO:H_2_O v/v ratio of 1:3, total flow 115 mL/min, and 4.3% CO.

The performance of PtNPs/SiO_2_ and PtNPs/CeO_2_/SiO_2_ catalysts in the WGS reaction as a function of temperature is presented in Figure 3. The CO_2_ formation rate of PtNPs/SiO_2_ confirmed the poor performance of Pt in the WGS reaction; at 250 °C, the rate was 0.3 mol_CO2_ mol_Pt_^–1^ min^–1^, increasing up to 1.6 mol_CO2_ mol_Pt_^–1^ min^–1^ at 400 °C. PtNPs/CeO_2_/SiO_2_, on the other hand, was much more active; there was a 14-fold increase at 250 °C compared to the PtNPs/SiO_2_ catalyst and about a 39-fold increase at 400 °C. It is well-known that CeO_2_ can greatly increase WGS reaction rates due to properties such as the availability of oxygen vacancies to activate water (redox properties) and reactive surface OH groups.^18^ Moreover, multiple works employing Pt/CeO_2_ as a catalyst associated the enhanced catalytic performance with the creation of interfacial Pt^δ+^–O_v_–Ce^3+^ sites as highly active sites.^12,18,35,40,45^

Structural and electronic characterization of PtNPs/SiO_2_ and PtNPs/CeO_2_/SiO_2_ (Figures 1 and 2 and Figure S2) evidenced that the supported PtNPs have similar size, shape, geometrical sites (i.e., WC, HUC, and UC Pt sites), and Pt oxidation state. The similarities between the catalysts allow a systematic study to evaluate the impact of the support (i.e., reducible or nonreducible) on the WGS reaction, emphasizing the role of Pt active sites and their relationship with surface intermediates and reaction pathways by *in situ* ME-DRIFTS coupled with PSD.

### 3.3 *In Situ* ME-DRIFTS

Initial measurements conducted under the sequential modulation of CO+H_2_O/He at 300 °C highlighted changes in the region assigned to chemisorbed CO over the Pt NPs as a function of time (Figure S7). The observed changes demonstrate that the particles’ electronic structure and geometrical sites evolved as a function of time. To fully assign the role of the Pt NPs geometrical sites and their correlation with the WGS reaction dynamics and mechanisms, a stable CO coverage needs to be achieved, with no further structural or electronic changes of the Pt NPs. By decreasing the temperature, it is expected that CO binds strongly to the surface of the Pt NPs and the conversion decreases, allowing for CO to cover the particles evenly along the entire measurements. Thus, ME-DRIFTS measurements at 250 °C were evaluated; however, the CO coverage was still impacted as a function of time (Figure S8a,b). The modulation of CO+H_2_O with CO was employed as a new strategy, keeping the fed CO partial pressure constant, and it was possible to observe that the CO coverage over the Pt NPs was kept constant (Figure S8c,d).

**Figure 4:**
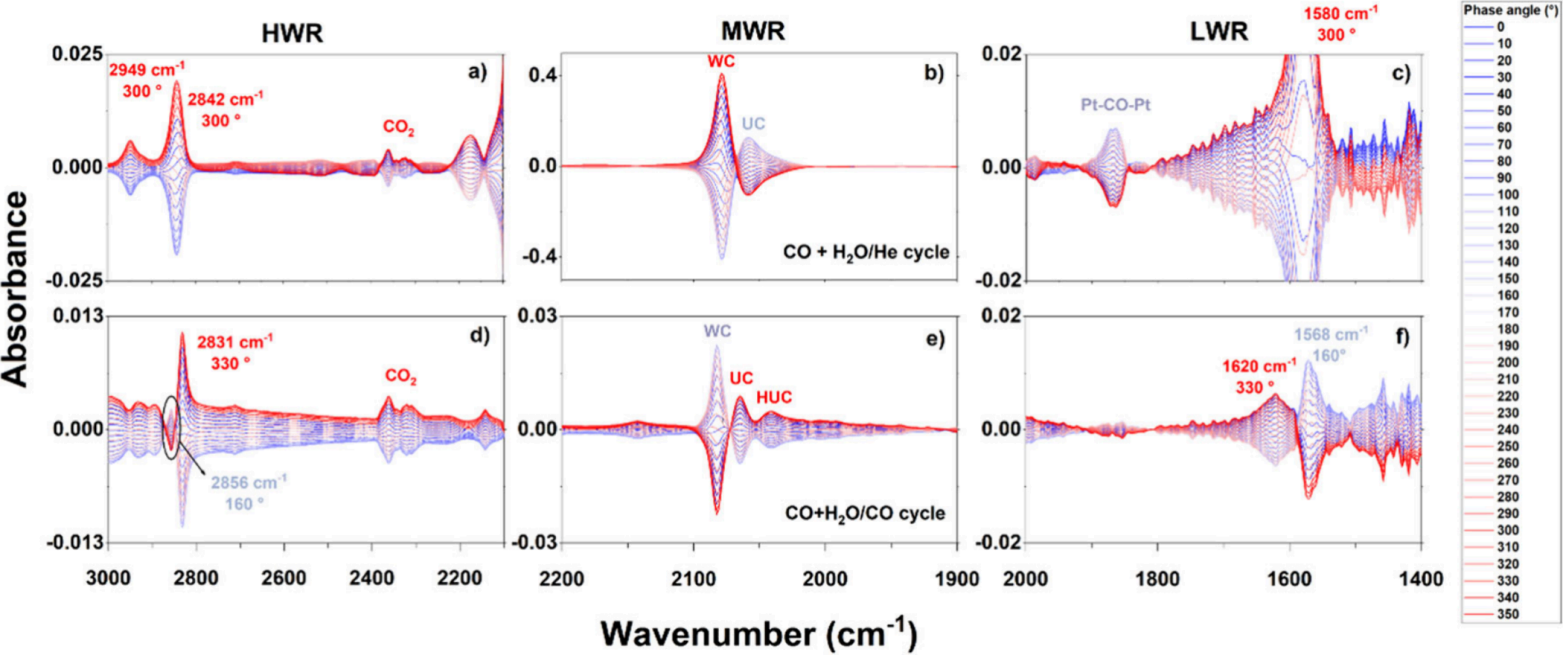
Phase domain spectra of *in situ* ME-DRIFTS applying PtNPs/CeO_2_/SiO_2_ at 250 °C obtained by reactants modulation in different conditions: (a,b,c) CO+H_2_O/He cycle, (d,e,f) CO+H_2_O/CO cycle, divided into three wavenumber regions: 3000–2200 cm^–1^ (HWR), 2200–1900 cm^–1^ (MWR), and 2000–1400 cm^–1^ (LWR).

The sequential modulations of CO+H_2_O/He and CO+H_2_O/CO were also evaluated by using *in situ* ME-DRIFTS measurements at 250 °C (Figure 4). For clarity, the spectra were divided into three wavelength regions: high (HWR, Figure 4a,d), middle (MWR, Figure 4b,e), and low (LWR, Figure 4c,f) wavenumber regions. By comparing the spectra from Figure 4, one can notice that the atmosphere affected the intermediates and phase responses. The HWR of the measurements modulated with He (i.e., CO+H_2_O/He, Figure 4a) presented two C–H stretching contributions (2949–2842 cm^–1^) from formate species with fast response (φ^PSD^ = 300°), in phase with CO_2_. The MWR highlights (Figure 4b) that the Pt geometrical sites had a fast response for WC sites (φ^PSD^ = 310°) and a slower response for UC sites (φ^PSD^ = 150°). At the LWR (Figure 4c), it was possible to observe a slow response related to bridged Pt–CO–Pt sites in phase with the UC sites and a fast response at ca. 1580 cm^–1^ related to formate species (φ^PSD^ = 300°).

The modulation of CO+H_2_O with CO (i.e., CO+H_2_O/CO) is shown in Figure 4d–f. At the HWR, it was possible to observe absorption bands from formate species, one responding faster (i.e., 2831 cm^–1^, φ^PSD^ = 330°) and the other slower (i.e., 2856 cm^–1^, φ^PSD^ = 160°), with the faster one in phase with CO_2_. Similar trends were observed for the LWR, with a faster and a slower response from the formate species, both presenting phase delays similar to that observed in the HWR. The Pt geometrical sites were also impacted, in which the WC sites were associated with a slower route (φ^PSD^ = 160°) and the UC and HUC sites with a faster one (φ^PSD^ = 330°). We highlight that the modulation with CO instead of He led to the inversion of WC and UC phase delays and resulted in active HUC sites and the absence of Pt–CO–Pt sites response. Considering that by modulating CO+H_2_O with CO the CO partial pressure was kept constant, a more realistic reactional condition was achieved. Thus, further experiments were conducted over the PtNPs/CeO_2_/SiO_2_ and PtNPs/SiO_2_ catalysts by modulating CO+H_2_O/CO at 250 °C.

**Figure 5:**
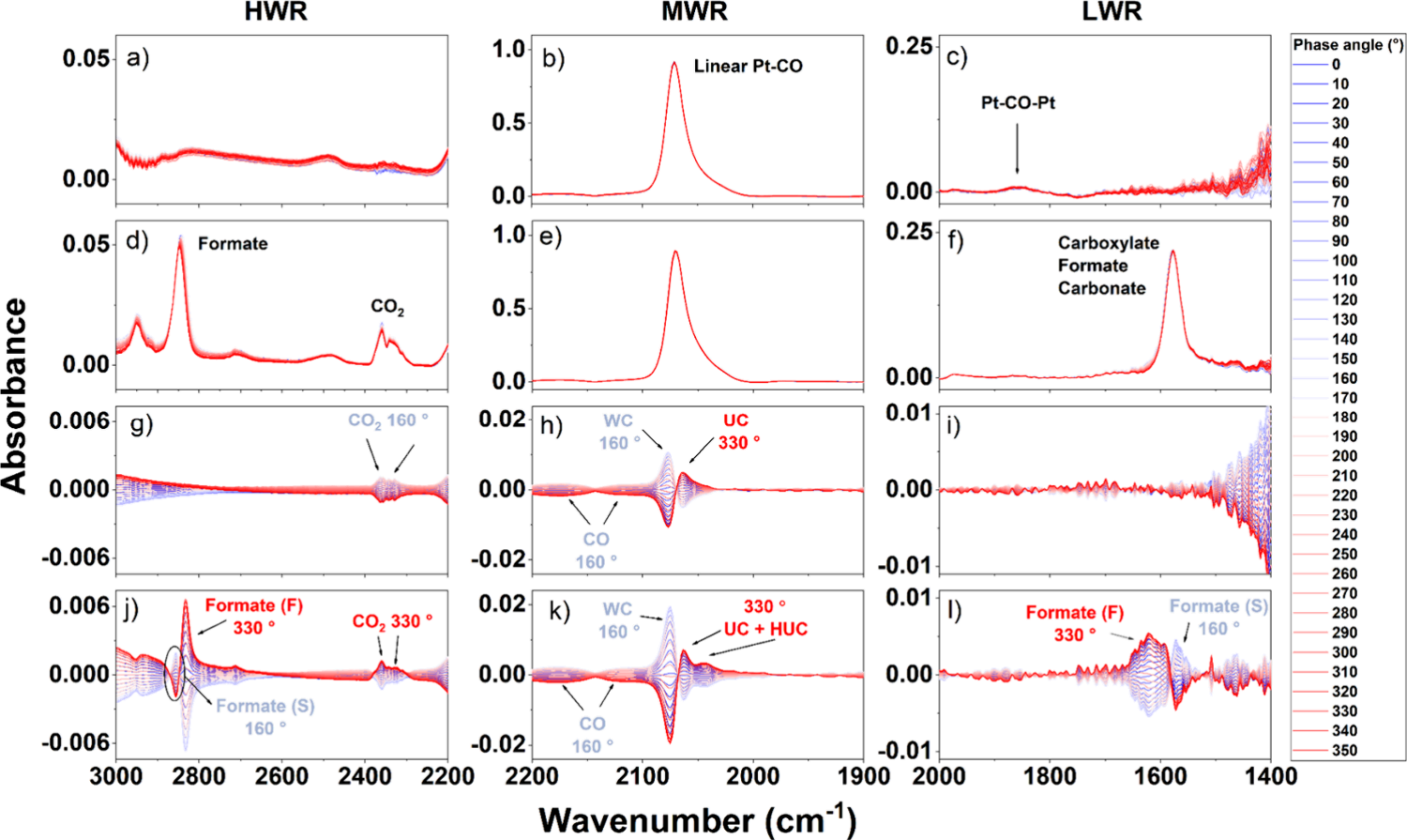
*In situ* ME-DRIFTS spectra obtained by reactants modulation (CO+H_2_O/CO) cycle at 250 °C, divided into three wavenumber regions: (a,d,g,j) 3000–2200 cm^–1^ (HWR), (b,e,h,k) 2200–1900 cm^–1^ (MWR), and (c,f,i,l) 2000–1400 cm^–1^ (LWR). Time domain: (a–c) PtNPs/SiO_2_, (d–f) PtNPs/CeO_2_/SiO_2_, and respective phase domain (g–i) PtNPs/SiO_2_ and (j–l) PtNPs/CeO_2_/SiO_2_.

*In situ* ME-DRIFTS spectra conducted under the sequential modulation of CO+H_2_O/CO at 250 °C are presented in Figure 5. Figure 5a–f presents the data in the time domain, and Figure 5g–l presents the data in the phase domain.

In the HWR, the bands corresponding to gas phase CO_2_ (2400–2300 cm^–1^) and formate species (*HCOO^–^, C–H stretching at 3000–2850 cm^–1^) were observed for PtNPs/CeO_2_/SiO_2_ (Figure 5d).^52,53^ These contributions were not clearly observed for PtNPs/SiO_2_ (Figure 5a). It is important to note that the presence of OH groups (from H_2_O and the support) caused an intense variation in the background in the HWR. In the MWR (Figure 5b,e), bands related to gas phase CO (2170 cm^–1^) and CO linearly bound to Pt (2070 cm^–1^) were observed in both samples. Differences arise again in the LWR. For PtNPs/CeO_2_/SiO_2_ (Figure 5f), bands assigned to O–C–O vibrations from carboxylates (*COO^–^), formates (*HCOO^–^), and carbonates (*CO_3_^δ−^) were present;^54−56^ for PtNPs/SiO_2_ (Figure 5c), the CO stretching band of bridged Pt-CO-Pt species (1950 cm^–1^) was the only one detected. Despite the information gathered from the time domain spectra in Figure 5a–f, it is not possible to distinguish among the entities adsorbed on the catalyst surface, i.e., formate, carboxylate, carbonates, linear CO-Pt, and bridged Pt-CO-Pt species, whether they were spectator species. For that, the phase domain spectra (Figure 5g–l) were analyzed.

For PtNPs/SiO_2_ (Figure 5g–i), the only evident contributions in the phase domain spectra were CO_2_ and CO in the gas phase (HWR and MWR), as well as linear Pt-CO adsorbed species (MWR). The bridged Pt–CO–Pt absorption band observed on the time domain spectra vanished, indicating that it did not respond to the modulation of the gases or was below the detection limit (it is important to remember the low activity of this catalyst). The phase domain spectra for PtNPs/CeO_2_/SiO_2_ (Figure 5j–l) give more information. They presented contributions corresponding to gas phase CO_2_ and CO (HWR and MWR) and adsorbed linear Pt–CO species (MWR). Additional contributions corresponding to formate (HWR from 3000 to 2800 cm^–1^ and LWR from 1700 to 1500 cm^–1^) were also observed. The broad band related to carbonates and carboxylates (observed on the time domain spectra, Figure 5f) was not evident in the phase domain spectra (Figure 5l). It is worth noting that the background signal in the HWR was affected by the water in the feed and the surface OH of the support, not allowing a direct comparison of the CO_2(g)_ evolution between the catalysts; this was also the reason that the identification of formate species was easier in the LWR.

Considering the phase-angle responses, in the Pt/SiO_2_ catalyst, CO adsorbed at WC sites was in phase with CO_2(g)_ formation (φ^PSD^ = 160°), Figure 5g,h, with a slower response to the modulation. The CO adsorbed at UC sites (φ^PSD^ = 330°), Figure 5h, responded quickly to the modulation, possibly involved in a faster and minor route. The phase-angle responses in the PtNPs/CeO_2_/SiO_2_ catalyst were richer. The *CO adsorbed on UC and HUC sites and the formation of formate (faster route, i.e., Formate (F)) and CO_2(g)_ were in-phase (φ^PSD^ = 330°), Figure 5j–l, suggesting that the CO_(g)_ activation occurred on the UC and HUC sites, followed by formate (F) formation, and its decomposition into CO_2(g)_. A more delayed response was observed for *CO adsorbed on WC sites and formate (slower route, i.e., formate (S)) formation (φ^PSD^ = 160°), suggesting that it corresponded to a slower route. The CO_(g)_ activation on UC and HUC sites (φ^PSD^ = 330°), followed by the migration of *CO to WC sites, could also be an alternative. The phase dependence of the main species observed for PtNPs/SiO_2_ and PtNPs/CeO_2_/SiO_2_, highlighting their synchronizm, is presented in Figures S11 and S12, respectively. Corroborated by the literature, the suppression of the carbonate band in the phase domain spectra confirms that this entity was a surface spectator.^57^ However, more detailed information about the participation of carboxylates in the reaction becomes inconclusive.

Mhadeshwar et al.^58^ demonstrated by DFT (density functional theory) that the WGS reaction on Pt can occur by (1) a one-step reaction mechanism, wherein the coupling between *CO and *H_2_O lead to the formation of carboxyl species and hydrogen (COOH* + H*), and further decomposition of *COOH into CO_2(g)_, or (2) the direct CO_2(g)_ formation (CO* + OH* ⇌ CO_2_* + H*), with the water dissociation as the rate-determining step.^59^ Stamatakis et al.^60^ demonstrated that the reactional feed composition can lead to structure-sensitive effects, directly impacting the preferred reaction pathway. For example, at a feed ratio of 0.5 (CO:H_2_O), the mechanism (1) was dominant, in which the *COOH formation was the rate-determining step, and its formation was primarily attributed to terrace sites; at lower CO:H_2_O ratios (10^–3^), mechanism (2) was dominant, in which water dissociation and CO oxidation involved the combination of interfacial and terrace sites. As the reaction conditions employed for the *in situ* ME-DRIFTS measurements presented a CO:H_2_O ratio of 0.3, mechanism (1) would be more favored. The lack of clear identification of intermediate species in the phase-domain spectra of PtNPs/SiO_2_ (Figure 5g–i) could be related to the very low activity of this catalyst. On the other hand, the bands observed in time domain spectra of PtNPs/CeO_2_/SiO_2_ indicate that not all formates and CO adsorbed on Pt participate in the reaction, remaining bound to the catalyst surface, in agreement with results reported by Kalamaras et al.^61^ and proposed by Aranifard et al.^40^

Regarding the nature of Pt sites involved in the reaction, the linearly Pt-bound CO band (Figure 5h,k) showed that the catalysts exhibited distinct responses. This means that the support nature (i.e., reducible or nonreducible) dictates how WC, UC, and HUC Pt sites contribute to the reaction. The MWR for each flow (CO+H_2_O and CO) is presented separately in Figure S9 for better analysis. Time domain spectra under CO+H_2_O (Figure S9a,b) and CO flows (Figure S9c,d) demonstrated that the contribution associated with adsorbed linear Pt–CO did not present significant changes upon steam feed. Hence, it can be assumed that the CO coverage was kept constant under both conditions, evidencing that a significant part of the CO adsorbed on Pt sites did not participate in the WGS reaction. In turn, the participation of CO bound to distinct Pt sites was clearly observed in the phase domain spectra: three components were observed for PtNPs/CeO_2_/SiO_2_ (Figure S9f), whereas for PtNPs/SiO_2_, only two were evident (Figures S9e, S10). For PtNPs/CeO_2_/SiO_2_, the three components are consistent with CO bound to Pt sites with different coordination (WC, UC, and HUC Pt sites, 2076, 2063, and 2044 cm^–1^, respectively), while for PtNPs/SiO_2_, the signal corresponding to HUC sites was not observed (Figures S10, S11), suggesting that CO poisoned these sites. Since the Pt NPs presented similar structural and electronic features in both catalysts (Figures 1 and 2), the presence of active HUC Pt sites for PtNPs/CeO_2_/SiO_2_ suggests the creation of Pt^δ+^–O_v_–Ce^3+^ in the metal–support interface, which directly impacted the catalytic performance (Figure 3). As observed in theoretical studies,^40^ HUC Pt sites would be the ones available to both interaction with ceria (corners) and exposure to reactants (CO and H_2_O), and only part of them would be in close contact with ceria. The participation of HUC Pt sites on PtNPs/CeO_2_/SiO_2_ would represent both the enhanced Pt activity caused by the interaction with ceria as well as the cleanup of these sites from CO poisoning, which is also a feature promoted by the interfacial sites.

Regarding the kinetic information that can be extracted from the phase domain spectra (Figure 5g–l and Figure S9e,f), it was observed that CO bound to low coordination Pt sites (UC and HUC) responded faster to the modulation, with a phase angle of φ^PSD^ = 330°, while WC Pt sites had an intense and more delayed response (φ^PSD^ = 160°). This suggests that all Pt sites (i.e., WC, UC, and HUC) may play a role in the WGS reaction mechanism for PtNPs/CeO_2_/SiO_2_ (only WC and UC for PtNPs/SiO_2_), but UC and HUC would have faster kinetics than WC, with adsorbed *CO being readily reactive in the presence of steam.

**Figure 6:**
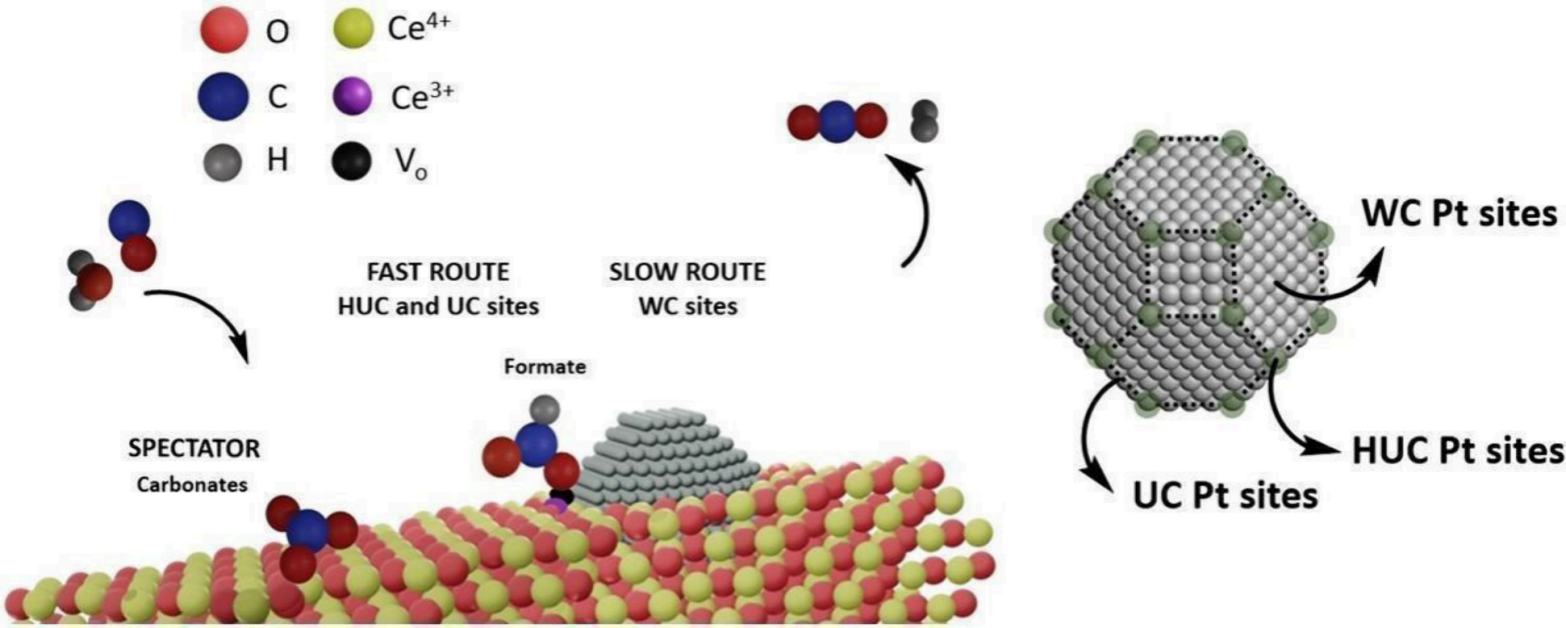
Schematic representation of the main intermediates species in WGS reaction for the PtNPs/CeO_2_/SiO_2_ system obtained by *in situ* ME-DRIFTS insights.

In summary, the analysis of the phase-angle responses for PtNPs/CeO_2_/SiO_2_ indicated that formate (F) is in-phase with CO bound to UC and HUC Pt sites (φ^PSD^ = 330°), while the formate (S) band had a delayed response in-phase with WC Pt sites (φ^PSD^ = 160°). The results showed that formate (F) and CO bound to UC and HUC Pt sites were the active intermediates from a faster path of the WGS mechanism, while CO bound to WC Pt sites and formate (S) species were the active intermediates of a slower reaction pathway. These results demonstrated that parallel reaction pathways with different kinetics occurred under the reaction conditions. A schematic representation of these findings is presented in Figure 6.

### 3.4 Insights and Limitations of ME-DRIFTS under WGS Reaction Conditions

ME-DRIFTS has been shown to be a powerful technique providing insights into surface species and adsorbates directly associated with the active metal sites and support under reaction conditions.^1−5^ This is particularly relevant in the case of WGS for distinguishing the differing roles of Pt when in contact with the support (i.e., reducible vs nonreducible). As shown in Figure 5, ME-DRIFTS measurements provided a better understanding of the contributions of the Pt geometrical sites and their correlation with the support reducibility and reaction intermediates in WGS, offering valuable insights that improve our knowledge of competitive mechanisms. We were not sensitive, however, to the participation of oxygen vacancies in our work, central to redox mechanisms. In this aspect, it is worth mentioning the work by Vecchietti et al.^52^ that correlated the oxygen vacancy formation with the Ce^3+^ infrared band at 2120 cm^–1^ on Pt/CeO_2_; however, the Ce^3+^ signal did not respond to the modulation of reactants in the ME-DRIFTS experiments. They concluded that the oxygen vacancies were fast filled, and the water activation was not the limited step. Due to the lack of information on oxygen vacancy in our experiments, we could not identify whether the formation of formate intermediates (i.e., fast and slow route) occurred by the classic “associative” or the “associative mechanism with redox regeneration” pathways. Aranifard et al.^40,62^ have demonstrated that the “associative carboxyl pathway with redox regeneration” would present higher reaction rates and lower activation barriers than the “associative carboxyl pathway” over the Pt/CeO_2_ catalyst. They suggested that the “associative carboxyl pathway with redox regeneration” and the redox mechanism could take place simultaneously; however, the former would have a dominant contribution. Accordingly, Kalamaras et al.^61^ also suggested that the participation of formate in the Pt/CeO_2_ catalyst would occur by the redox regeneration pathway. Thus, we could expect that for PtNPs/CeO_2_/SiO_2_ the “associative formate with redox regeneration” and the classical associative pathways could be taking place in an independent or simultaneous regime. Our hypothesis is supported by Meunier et al.^33^ which performed studies based on SSITKA (Steady-State Isotopic Transient Kinetic Analysis) with a 2 wt % Pt/CeO_2_. At 160 °C, formate was practically an inactive entity, becoming an actual reaction intermediate at 220 °C. When studying an Au/Ce(La)O_2_ catalyst, Meunier et al.^63^ identified the participation of both fast and slow formate species through the SSITKA technique, similar to observations of the current work. Vecchietti et al.^52^ also showed that formate species were involved in the WGSR reaction through a minor or slower route, while carboxylate and carboxyl species participated in a faster route, in-phase with CO_2_ formation in Au/CeO_2_. It has been discussed in the literature that the kinetic importance of surface species could be dramatically impacted in a narrow temperature range, and therefore caution is required when attempting to generalize the reaction mechanism based on data using different reaction temperatures, feed compositions, or even differently prepared and pretreated catalysts (e.g., distinct calcination and reduction conditions). Thus, formate species were reported to switch from inactive species at 160 °C to active intermediates at 220 °C for Pt/CeO_2_ under the given reaction conditions.^63^ Kalamaras et al.^61^ also suggested that formate species would be active intermediates in the WGS reaction conducted over Pt/CeO_2_ catalysts at 300 °C; however, quantitative measurements evidenced that the route involving formate would have a minor role in the activity due to the dominance of the redox pathway. The authors also proposed that the redox mechanism would be faster than the associative path (i.e., formate) and that part of the adsorbed CO and formate entities would not participate in the overall WGS reaction. Hence, the dominant reaction pathway over Pt/CeO_2_ catalysts may depend strongly on the reaction conditions, which also dictates the role of formate intermediates.

Finally, Ziemba et al.^14^ investigated the WGS reaction using ME-DRIFTS on Cu/CeO_2_ at 190 °C, highlighting the impact of maintaining the H_2_O and CO partial pressures constant (i.e., H_2_O/H_2_O+CO and CO/H_2_O+CO). While keeping the H_2_O partial pressure constant, the WGS reaction was governed by a redox mechanism, in which H_2_O was activated and cleaved on oxygen vacancy sites, thus regenerating the lattice oxygen site of the support. The presence of CO in the reactants modulation acted as the reducing agent, responsible for generating new oxygen vacancies and converting CO to CO_2_. On the other hand, they demonstrated that maintaining constant CO partial pressure was crucial for probing associative reaction pathways, in which it was possible to observe the generation of reaction intermediates (e.g., formates, carbonates). This example shows that the reactants modulation impacts the preferred reaction pathways and intermediate formation under WGS reaction conditions. As the focus of our work was probing the impact of Pt geometrical active sites on the WGS reaction, keeping the CO partial pressure was a crucial step in keeping the CO coverage over the Pt NPs constant, which limited this type of analysis.

## 4 Conclusions

The impact of different Pt geometrical sites on the WGS reaction according to the reducibility of the support was demonstrated. PtNPs/SiO_2_ (nonreducible) and PtNPs/CeO_2_/SiO_2_ (reducible) catalysts were produced using the same premade colloidal NPs. Both catalysts presented PtNPs with similar size, shape, geometrical sites (i.e., WC, HUC, and UC Pt sites), and Pt oxidation states, allowing a systematic study to shed light on the role of the support over the catalytic activity. The WGS reaction rate evidenced the impact of the reducibility of the support by increases of 14-fold at 250 °C and about 39-fold at 400 °C when compared to the nonreducible support.

A detailed analysis by *in situ* ME-DRIFTS coupled with PSD provided important insights into the Pt geometric active sites and their relationship with surface intermediates. For PtNPs/CeO_2_/SiO_2_, CO bound to UC and HUC Pt sites and formate (F) species were the active intermediates from a faster pathway of the WGS mechanism (φ^PSD^ = 330°), while CO bound to WC Pt sites and formate (S) species were the active intermediates of a slower reaction pathway (φ^PSD^ = 160°). Carbonate was associated with spectator surface species. For the PtNPs/SiO_2_ catalyst, CO adsorbed at WC sites was in phase with CO_2(g)_ formation (φ^PSD^ = 160°), with a slower response to the modulation. These results demonstrated that parallel reaction pathways with different kinetics occurred under the employed reaction conditions, and the support nature (i.e., reducible or nonreducible) was responsible for dictating how WC, UC, and HUC Pt sites were contributing to the reaction pathways. It is worth mentioning, however, that we were not sensitive to oxygen vacancy and redox mechanisms.

This work showed that the strong binding of intermediates can poison low-coordination Pt sites and that an effective interface is crucial to promote the availability of these active sites, promoting enhanced WGS activity. The coupling of *in situ* ME-DRIFTS time and phase domain spectra has demonstrated its capability to distinguish surface-active intermediates from spectator species, providing kinetic information, as evidenced by the phase angle response of each species. Moreover, it was possible to show the effective participation of distinct Pt geometrical sites (i.e., HUC, UC, and WC) according to the reducibility of the support.

## Supporting Information Available

Experimental procedures; XANES and EXAFS data on PtNPs/CeO_2_/SiO_2_; XRD and TEM of CeO_2_NPs; time domain DRIFTS spectra of the modulation of (CO+H_2_O/He) at 300 °C for PtNPs/SiO_2_ and PtNPs/CeO_2_/SiO_2_; time and phase domain DRIFTS spectra of Pt-CO wavenumber region for PtNPs/SiO_2_ and PtNPs/CeO_2_/SiO_2_ at 250 °C; and phase domain DRIFTS spectra of the modulation of (CO+H_2_O/CO) at 250 °C for PtNPs/SiO_2_ (PDF)
