# Supplementary material for: Insights from Modulation-Excitation Spectroscopy into the Role of Pt Geometrical Sites in the WGS Reaction
Source: ACS Appl Mater Interfaces. 2025 Feb 22;17(9):13221–31. doi: 10.1021/acsami.4c21397 (PMC11891839; doi:10.1021/acsami.4c21397)
Supplement: Supplementary file 1 — am4c21397_si_001.pdf [file am4c21397_si_001.pdf]

Support Information

Insights from Modulation-Excitation Spectroscopy into the role of Pt geometrical sites in the WGS reaction

Tathiana M. Kokumai[1], Larissa E. R. Ferreira[1], Guilherme B. Strapasson[1,2], Lea Pasquale[3], Liberato Manna[3], Massimo Colombo[3], and Daniela Zanchet[1]*

[1]*Institute of Chemistry, University of Campinas, Campinas, SP, 13083-970 (Brazil)*

[2]*Brazilian Synchrotron Light Laboratory, CNPEM, Campinas, SP, (Brazil)*

[3]*Nanochemistry Dept., Italian Institute of Technology, Genoa, GE, 16163 (Italy)*

*zanchet@unicamp.br

EXPERIMENTAL

Characterization

The PSD procedure allows the observation of IR active species signals with enhanced signal-to-noise ratio with respect to the time-resolved spectra and filters out all contributions of signals not responding with the same frequency as that of the stimulation (in this case, the variation of the gas feed). The collected average time-resolved spectra , which would be response A(t) obtained by MES, were mathematically processed using Matlab® software to extract the kinetic information, i.e., phase domain spectra, by means of the PSD method according to the following equation:

$$A_k(\varphi_k^{PSD}) = \frac{2}{T}\int_0^T A(t) sin(k\omega t + \varphi_k^{PSD})dt \qquad \text{(Equation 1)}$$

where T is the length of one period, ω is the modulation frequency, k is the demodulation index, φ is the demodulation phase angle for kω demodulation, and A(t) and A_k are the active species response in time- and phase-domain, respectively.[1-6]

Since the stimulation is a periodic function, the frequency of a full modulation cycle is related to a 360 ° phase angle (which corresponds to a 0° phase delay). Analysis of the phase angle (0°–360°), i.e., time-delay of reaction intermediates, provides kinetic information of chemical species involved in the surface processes. Each active species will respond to the periodic stimulation (gas changes) with a given phase angle (from 0

to 360 °) and will then present a phase delay in relation to the corresponding stimulation frequency. The species showing high phase angles, for example, 330 °, respond fast to the stimulation, with a phase delay of only 30 °, while species with low phase angles, 160 °, respond more slowly, with a delay of 200 ° in relation to the modulation frequency.[2,7] The intensity of the signals in the phase domain spectra is related to the magnitude of the change caused by the perturbations in the concentration of the feed gas.

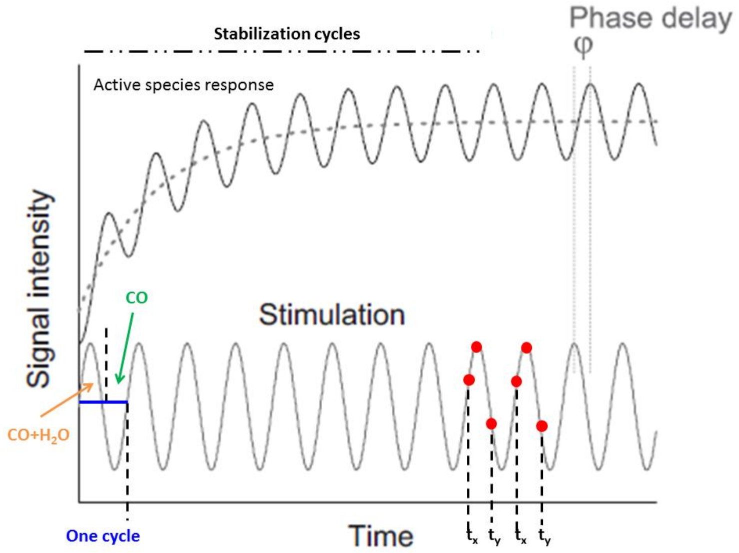

Figure S1: Illustration of an MES experiment showing the periodic stimulation (modulation) of a system and the corresponding response of active species (Adapted from [2]).

Pt-L3 edge (11564 eV) X-ray absorption fine structure spectra (XAFS) were acquired at XAFS2 beamline at Brazilian Synchrotron Light Laboratory (LNLS-CNPEM). The data were collected in transmission mode, using a Si (111) monochromator. The spectra of Pt foil and PtO_2 references were acquired for comparison. XANES and EXAFS data analysis were performed with Athena and Artemis codes within the Demeter package following the standard procedures for alignment, normalization and background removal[8]. Figure S2 shows the XAFS data. Pt metallic dispersion was estimated by the structural data obtained by EXAFS (extended X ray absorption fine structure) analysis (Table S1) following the work by Calvin et al.[9]

RESULTS

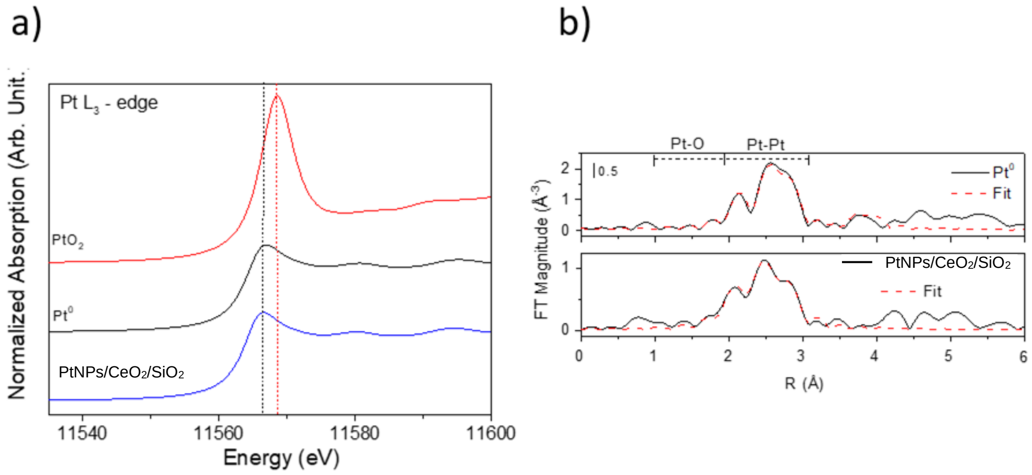

Figure S2: a) XANES spectra at Pt-L3 edge and b) Fourier transform of EXAFS oscillations for the catalyst PtNPs/CeO_2/SiO_2 after reduction (100 mL/min, 5%H_2/He, 400 °C, 1h) and the best fits for the first coordination shell. The results for the Pt^0 reference are presented for comparison. Vertical dashed lines indicate the fitting range (1.3-3.3 Å) for all samples.

Table S1: Structural parameters of Pt domains for PtNPs/CeO_2/SiO_2 obtained by EXAFS analysis (CN - coordination number; R - interatomic distance; σ^2 – Debye Waller factor).

	Path	CN	R (Å)	σ^2(Å^2)	R-factor	Dispersion (%)
Pt foil (reference)	Pt-Pt	12	2.769 (0.003)	0.005 (0.001)	0.007	-
PtNPs/CeO_2/SiO_2	Pt-Pt	8.9 (0.6)	2.743 (0.004)	0.007 (0.001)	00.7	69
	Pt-O_L[b]	1.6 (0.6)	3.205 (0.028)	0.007 (0.001)		

[a] fixed, according to fcc structure of bulk Pt.

[b]Pt-OL stands for a long Pt-O bond, longer than the one found for Pt-O first nearest neighbors in PtO_2.

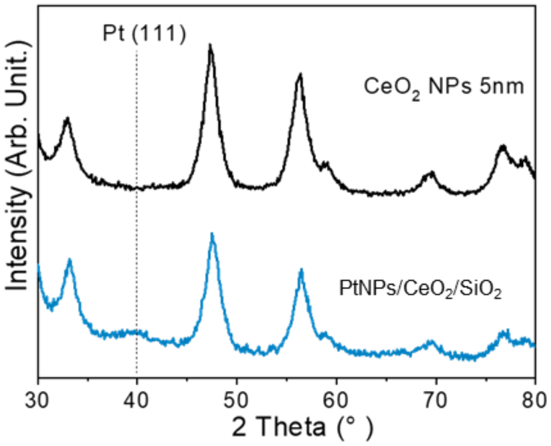

Figure S3: XRD patterns of 5 nm CeO_2 NPs and $PtNPs/CeO_2/SiO_2$.

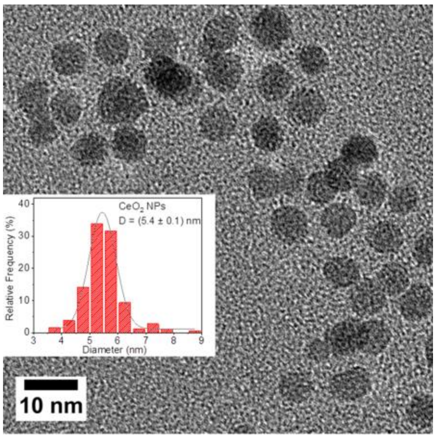

Figure S4: TEM images and size distributions of colloidal CeO_2 NPs 5 nm.

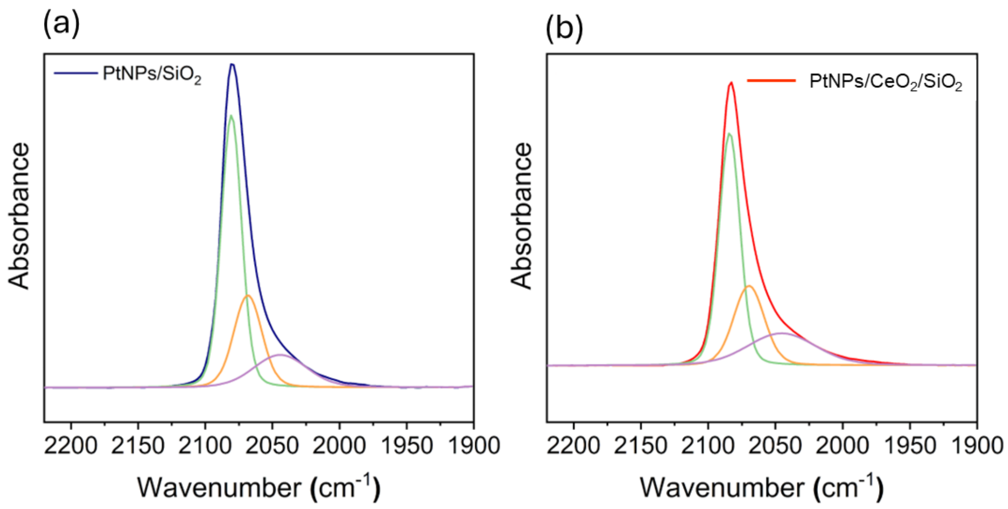

Figure S5: Deconvoluted CO-DRIFTS spectra of (a) PtNPs/SiO_2 and (b) PtNPs/CeO_2/SiO_2 after exposure to CO flow (1% CO/He v/v) for 10 min at room temperature.

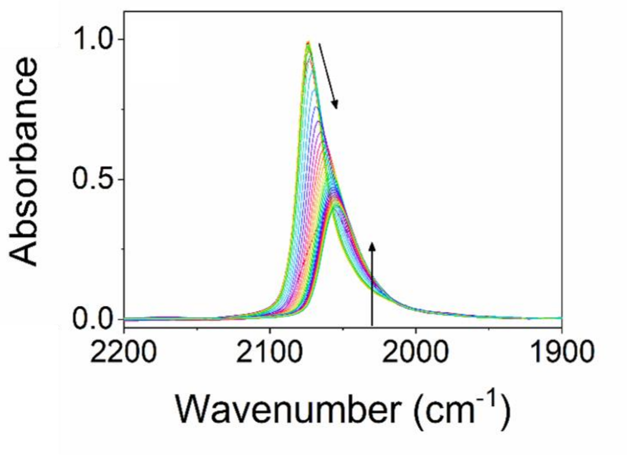

Figure S6: Time domain spectra during reactants modulation (CO+H_2O/He) cycle at 300 °C, shown in the MWR region (2200-1900 cm^{-1}) for and PtNPs/CeO_2/SiO_2.

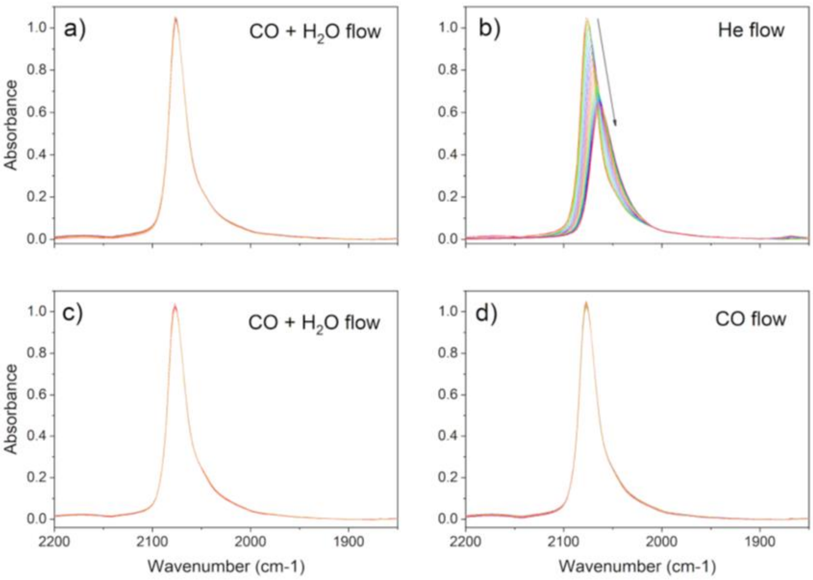

Figure S7: Time domain spectra during reactants modulation CO+H_2O/He (a-b) and CO+H_2O/CO (c-d) at 250 °C, shown in the MWR region (2200-1900 cm^{-1}) for PtNPs/CeO_2/SiO_2.

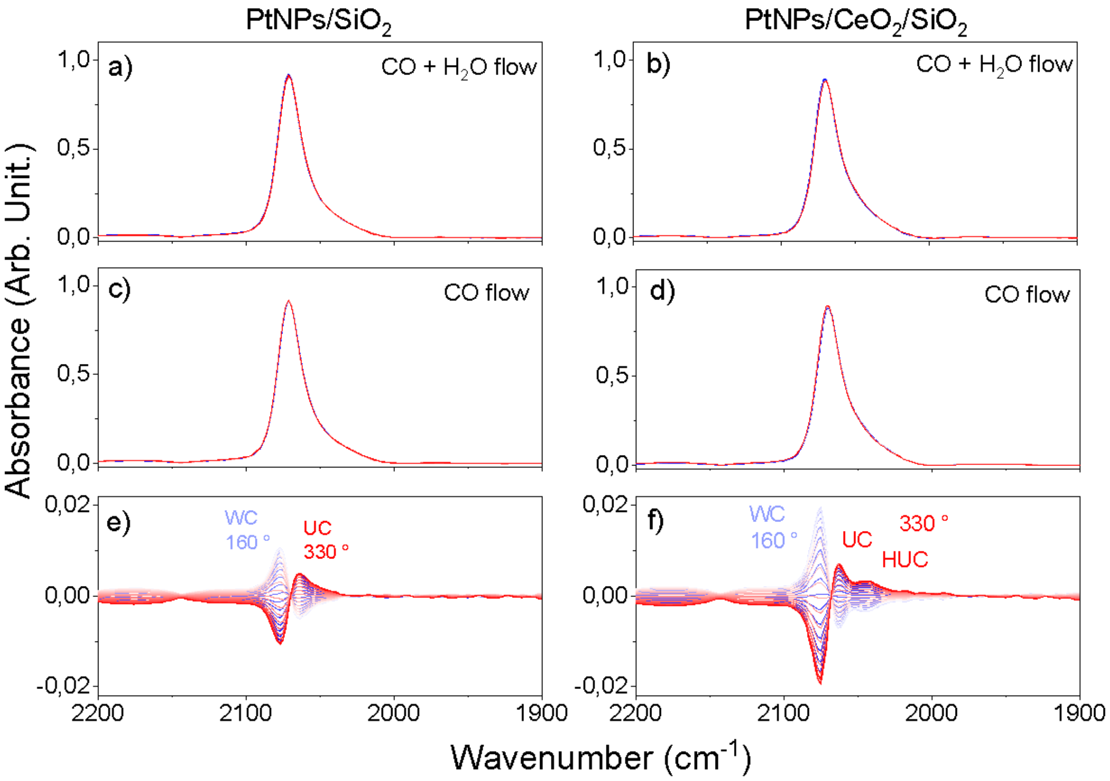

Figure S8: Comparison of time domain spectra at the Pt-CO wavenumber region for PtNPs/SiO_2 (left) and PtNPs/CeO_2/SiO_2 (right) during CO+H_2O (a,b) and CO (c,d) modulation flows at 250 °C and the corresponding phase domain spectra (e,f).

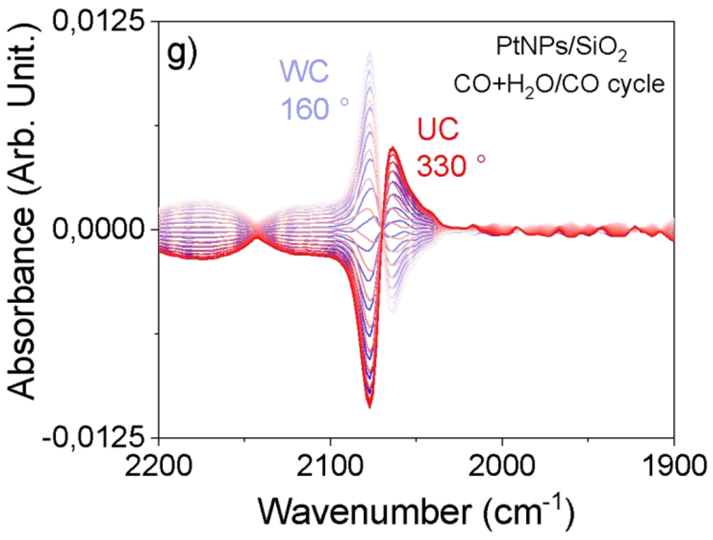

Figure S9: Phase domain spectra for PtNPs/SiO_2 during CO+H_2O/CO modulation flows at 250 °C, at a smaller scale on y axis to highlight HUC Pt sites region.

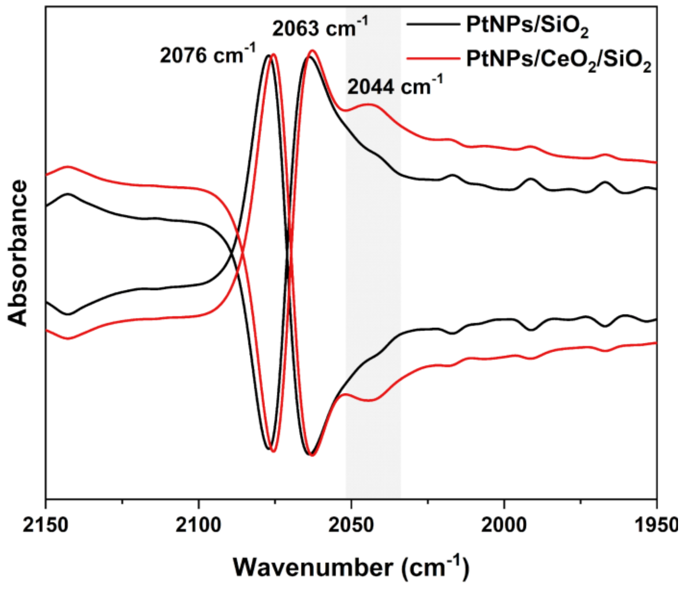

Figure S10: Phase domain of selected spectra for PtNPs/SiO_2 and PtNPs/CeO_2/SiO_2 during CO+H_2O/CO modulation flows at 250 °C highlighting the presence of the three Pt geometrical components.

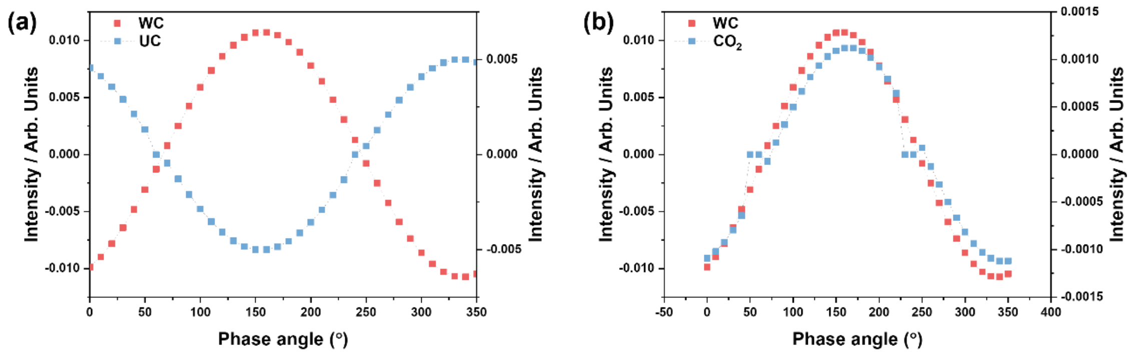

Figure S11: Phase dependence on CO_2 and chemisorbed CO species for PtNPs/SiO_2 catalyst during CO+H_2O/CO modulation flows at 250 °C.

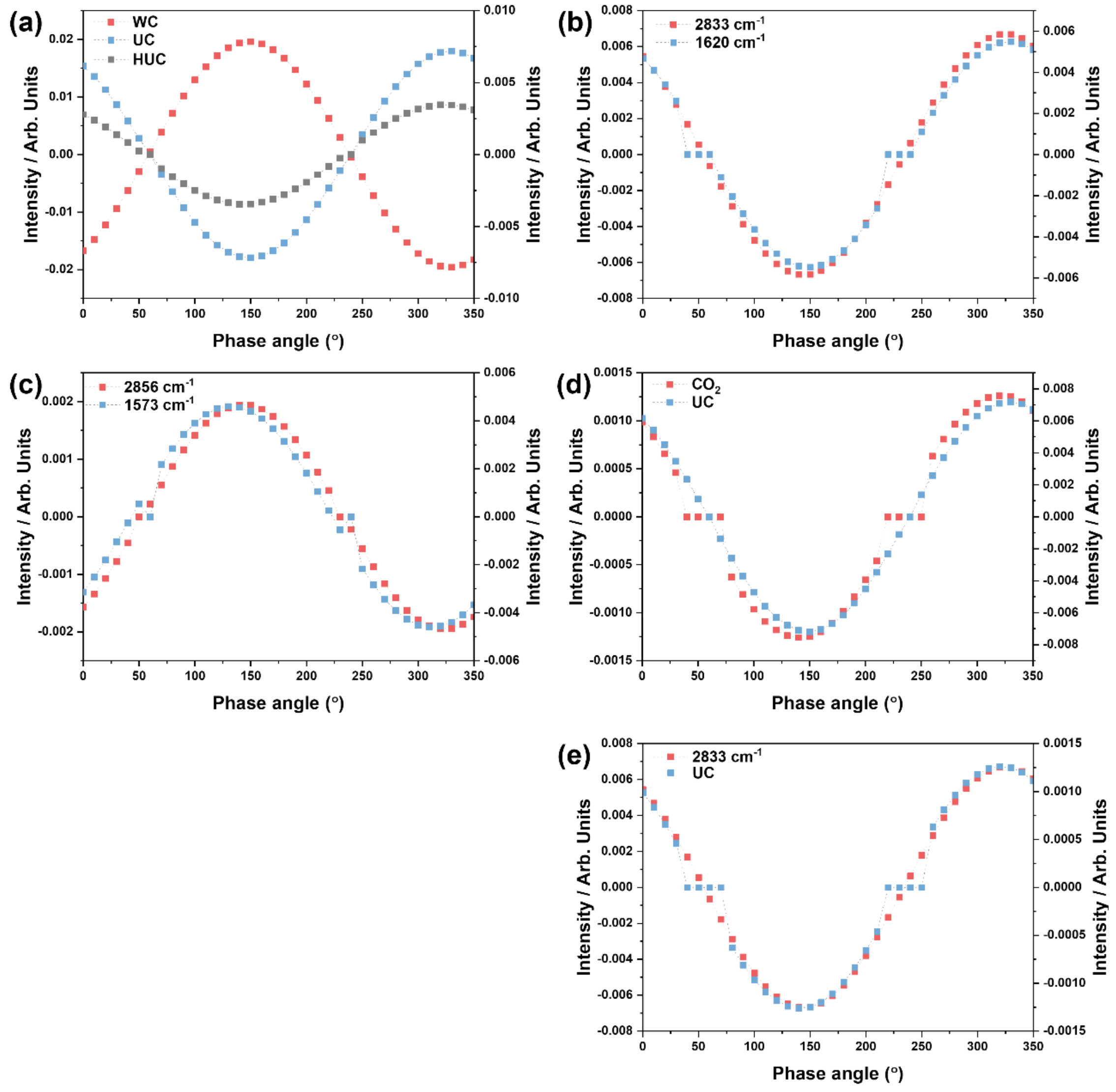

Figure S12: Phase dependence on formate, CO_2, and chemisorbed CO species for PtNPs/CeO_2/SiO_2 catalyst during CO+H_2O/CO modulation flows at 250 °C.

REFERENCES

1. Bürgi, T. & Baiker, A. In situ infrared spectroscopy of catalytic solid-liquid interfaces using phase-sensitive detection: Enantioselective hydrogenation of a pyrone over Pd/TiO_2. *J. Phys. Chem. B* **106**, 10649–10658 (2002).

2. Urakawa, A., Bürgi, T. & Baiker, A. Sensitivity enhancement and dynamic behavior analysis by modulation excitation spectroscopy: Principle and application in heterogeneous catalysis. *Chem. Eng. Sci.* **63**, 4902–4909 (2008).

3. Aguirre, A. & Collins, S. E. Selective detection of reaction intermediates using concentration- modulation excitation DRIFT spectroscopy. *Catal. Today* **205**, 34–40 (2013).

4. Müller, P. & Hermans, I. Applications of Modulation Excitation Spectroscopy in Heterogeneous Catalysis. *Ind. Eng. Chem. Res.* acs. (2017).

5. Zaera, F. New advances in the use of infrared absorption spectroscopy for the characterization of heterogeneous catalytic reactions. *Chem. Soc. Rev.* **43**, 7624–7663 (2014).

6. Kydd, R. *et al.* Temperature-induced evolution of reaction sites and mechanisms during preferential oxidation of CO. *J. Catal.* **277**, 64–71 (2011).

7. George, C. *et al.* CO Oxidation on Colloidal $Au_{0.80}Pd_{0.20}$ –Fe_xO_y Dumbbell Nanocrystals. *Nano Lett.* **13**, 752–757 (2013).

8. Ravel, B.; Newville, M. ATHENA, ARTEMIS, HEPHAESTUS: Data Analysis for X-Ray Absorption Spectroscopy Using IFEFFIT. J. Synchrotron Radiat. 2005, 12 (Pt 4), 537– 541.

9. Calvin, S.; Riedel, C. J.; Carpenter, E. E.; Morrison, S. A.; Stroud, R. M.; Harris, V. G. Estimating Crystallite Size in Polydispersed Samples Using EXAFS. Phys. Scr. 2005, 2005 (T115), 744..
